# Impact of outpatient preanesthetic consultation on perioperative outcomes in 700 urological patients in a tertiary hospital: a retrospective observational study^☆^

**DOI:** 10.1016/j.bjane.2026.844743

**Published:** 2026-03-06

**Authors:** Silvana Lellouche de Castro, Alexandra Rezende Assad, Aline d’Avila, Camila Spiller, René Murilo de Oliveira, Ismar L. Cavalcanti, Nubia Verçosa

**Affiliations:** a Universidade Federal do Rio de Janeiro, Postgraduate Programme in Surgical Sciences, Department of Surgery, Rio de Janeiro, RJ, Brazil; b Universidade Federal Fluminense, Department of General and Specialized Surgery, Niterói, RJ, Brazil; c Universidade do Estado do Rio de Janeiro, Institute of Geography, Cabo Frio, RJ, Brazil; d Americas' Medical City, Division of Anaesthesiology, Rio de Janeiro, RJ, Brazil; e Universidade Federal do Rio de Janeiro, Hospital Universitário Clementino Fraga Filho, Division of Urology, Rio de Janeiro, RJ, Brazil; f Universidade Federal Fluminense, Postgraduate Programme in Medical Sciences, Department of General and Specialised Surgery, Anaesthesiology, Niterói, RJ, Brazil

**Keywords:** Ambulatory care, Observational study, Perioperative medicine, Preoperative care, Urological diseases

## Abstract

**Background:**

Outpatient preanesthetic consultation improves patient assessment and anesthetic planning, enhancing safety and reducing complications. Conducted in advance, it allows systematic, individualized planning. Uncontrolled hypertension, common in 60% of people over 60, is a major cause of surgery cancellation and increases cardiovascular risk. This study evaluates its impact on preventing perioperative complications and optimizing clinical and surgical outcomes in urological patients.

**Methods:**

This retrospective observational study analyzed 700 patients (≥ 18 years) who attended outpatient preanesthetic consultation before urological surgery. Clinical conditions, systemic blood pressure, heart rate was analyzed during outpatient, preoperative, and intraoperative periods. Preoperative and intraoperative complications were recorded to evaluate consultation impact.

**Results:**

Among 700 patients (89.6% male, mean age 64.2), ASA II was most common classification. Hypertension was identified in 53.7% of patients during outpatient evaluation. All hypertensive patients received antihypertensive treatment until surgery, with blood pressure maintained within normal limits. Preoperative findings in the operating room included hypertension (2.0%), anemia (0.57%), atrial fibrillation (0.14%), and asthma (0.14%). Intraoperative events included hypertension (2.28%), hypotension (8.14%), bradycardia (1.14%), and inadequate neuraxial block (2.85%). One surgery was canceled due to hypertension. Blood pressure significantly decreased preoperatively and intraoperatively compared to outpatient values (p < 0.0001). Heart rate also decreased significantly intraoperatively. This single-center study has limitations, including absence of comparison groups assessed by other specialties or only day of surgery.

**Conclusion:**

Reductions in systolic and diastolic blood pressure were documented upon operating room entry among patients evaluated in outpatient preanesthetic consultation, with a very low surgery cancellation rate.

## Introduction

The outpatient preanesthetic consultation was first proposed in 1949 by Alfred Lee, who emphasized its role in preventive medicine. He noted many patients arrived for surgery in inadequate clinical condition, avoidable with earlier preparation.^1^ A preoperative assessment conducted days before surgery is essential to understand clinical profile, perform evaluations, plan anesthesia, and educate patients. Its main goals are to reduce morbidity and mortality, improve awareness, and alleviate anxiety.^2^, ^3^, ^4^, ^5^ Preoperative education reduces anxiety across all age groups, highlighting the value of tailored preanesthetic information.^6^^,^^7^

Beyond preparation and education, outpatient preanesthetic consultation plays a central role in perioperative care by enabling early risk assessment and identification of comorbidities that may affect outcomes. More than a screening opportunity, it is also a moment for clinical adjustment of known or newly diagnosed conditions, contributing to safety and reducing complications.

A brief preanesthetic visit shortly before surgery does not replace a comprehensive assessment. In Brazil, Resolution n° 2.174/2017 of the Federal Council of Medicine recommends anesthesiologists know the patient’s clinical condition before surgery. Assessment should be performed on an outpatient basis, except in urgent or emergency cases, when the anesthetist evaluates the patient immediately before entering the operating room. Preoperative evaluations by clinicians or cardiologists, while important, are not sufficient to address anesthetic-specific risks. The resolution requires anesthesia-specific informed consent before the procedure.^8^

Practice guidelines were adopted in France in 1994^9^ and by the American Society of Anesthesiologists in 2001.^10^ In countries like the USA, clinics exist solely for preoperative evaluation, with surgeons referring patients to anesthesiologists and nurses.^10^ Many hospitals, including Brazil, use telemedicine for preanesthetic assessments, increasing convenience and satisfaction while reducing costs. Studies show telemedicine does not increase surgery cancellations, maintaining quality and safety.^11^, ^12^, ^13^

Shared decision-making has emerged, combining patients’ preferences with medical expertise to determine personalized care. Perioperative professionals must develop guidelines and interventions promoting meaningful dialogue.^14^

In surgical specialties such as urology, where many procedures are elective and patients often have chronic conditions, outpatient consultation is essential. Common comorbidities ‒ hypertension, uncontrolled diabetes, chronic respiratory diseases, obesity, and tobacco use ‒ are risk factors for perioperative complications, including hemodynamic instability, delayed recovery, and infections. Inadequate preparation increases intra- and postoperative risks, while early management improves safety and outcomes.^15^

This study hypothesizes that outpatient preanesthetic consultation is the most appropriate method for preparing patients for surgery, potentially reducing perioperative complications and ensuring greater safety in urological procedures. The general objective was to demonstrate impact on preventing complications and optimizing clinical and surgical outcomes. Specific objectives were: (1) Describe baseline patient characteristics; (2) Assess cardiovascular abnormalities; (3) Analyze systemic blood pressure and heart rate across outpatient, preoperative, and intraoperative periods; and (4) Identify preoperative and intraoperative complications.

## Methods

This retrospective observational study was based on the review of medical records of patients who underwent surgical procedures at Clementino Fraga Filho University Hospital (HUCFF), Federal University of Rio de Janeiro, Brazil. A convenience sample of 700 patients who underwent urological surgeries and completed outpatient preanesthetic consultation was included. These patients were selected from a broader surgical population involving multiple specialties.

Consultations at the Preanesthetic Outpatient Clinic (PAC) were conducted by anesthesia residents and medical students under the supervision of an anesthesiology professor. During these evaluations, patients received guidance on anesthetic techniques and intraoperative monitoring.

The study was approved by the HUCFF Research Ethics Committee (CAAE: 3973.3020.0.0000.5257) on February 2, 2021, and registered in the Brazilian Registry of Clinical Trials (ReBEC n° 3vxknd7) on August 10, 2022. It followed the Declaration of Helsinki and STROBE guidelines for observational studies. The Free and Informed Consent Form (FICF) was waived.

**Figure 1:**
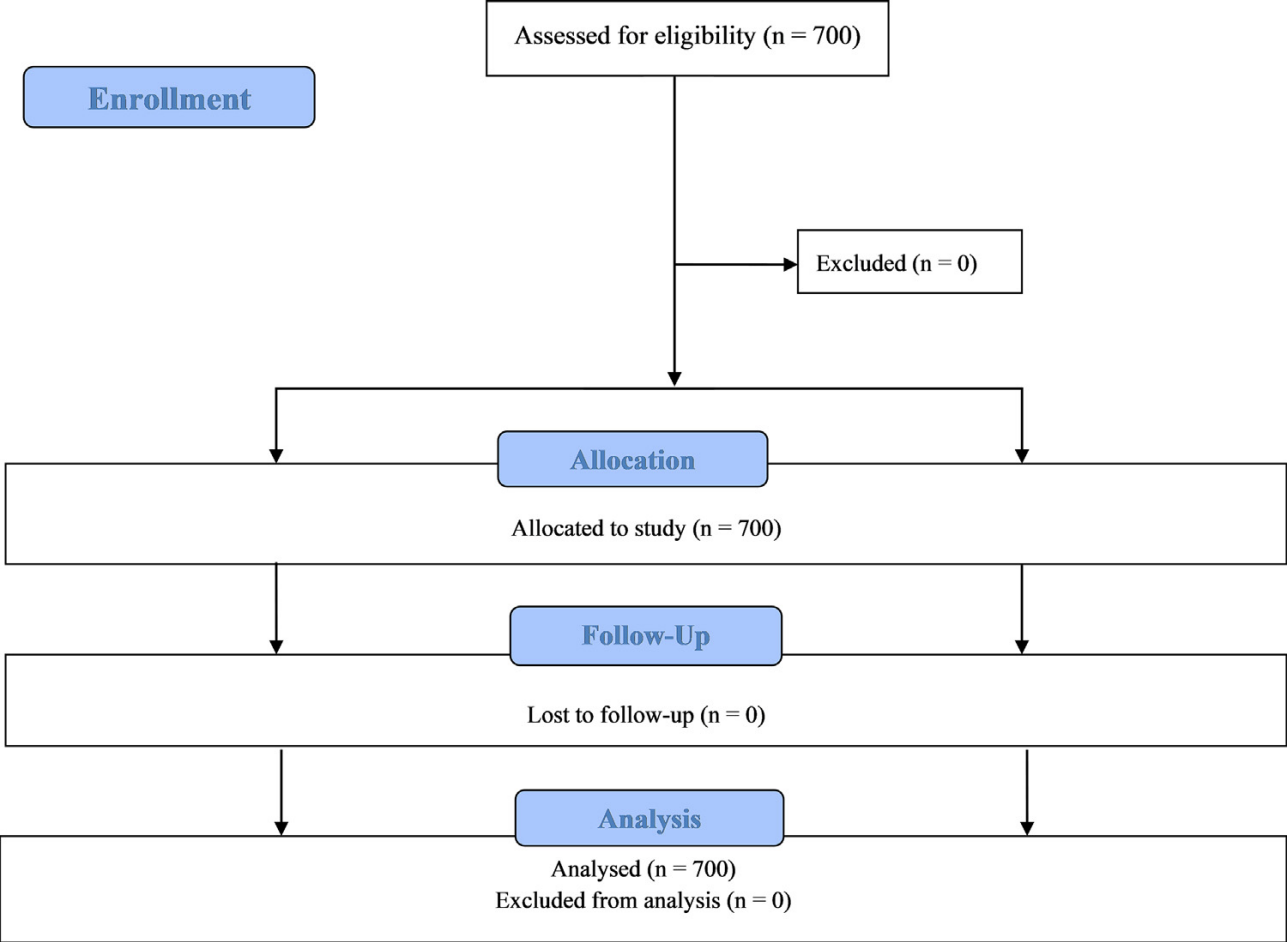
Flowchart of patient selection and inclusion.

The inclusion criteria were patients over 18 years of age, of both sexes, and classified as ASA physical status I to III. No patients were excluded from the study (Fig. 1). Data were collected from HUCFF medical records (2022–2023), including outpatient Preanesthetic Consultation (PAC) forms, anesthesia and surgical reports, and documentation of preoperative and intraoperative complications. Baseline variables included age, sex, ethnicity, weight, height, and ASA physical status. Electrocardiography (ECG) and Echocardiography (ECHO) results were also recorded.

Information on comorbidities was obtained from clinical history, physical examination, and complementary tests, and was used to assess their frequency and relevance for anesthetic risk stratification. All data were collected according to a predefined study protocol and subsequently analyzed using appropriate statistical methods.

The preoperative period began with the patient's admission to the operating room for monitoring and venous access. Blood pressure and other physiological measurements were performed by anesthesiology staff and residents, trained in standardized measurement techniques. Monitoring was conducted using a Narcosul multiparametric monitor for all patients, including five-lead electrocardiography, pulse oximetry, noninvasive blood pressure, and capnography, following institutional protocols. Standardization of equipment and measurement procedures was adopted throughout the study to ensure consistency and reliability of collected data. The intraoperative period extended from anesthesia induction or anesthetic blockade to the end of the surgical procedure. Blood pressure and heart rate were recorded at three defined points: during the outpatient preanesthetic consultation and upon admission to the operating room (preoperative). Intraoperatively, three measurements were taken: at the beginning, middle, and end of surgery, and their average was used for analysis. Hypertension was defined as blood pressure > 140/90 mmHg, tachycardia as heart rate > 100 bpm, hypotension as blood pressure < 90/60 mmHg, and bradycardia as heart rate < 60 bpm.

As a retrospective study, no standardized anesthetic protocol was applied. The anesthetic technique was selected by the attending anesthesiologist, based on the patient’s clinical condition and the surgical procedure. The distribution of techniques is detailed in the results. Complications were recorded during both preoperative and intraoperative periods. The study protocol remained unchanged throughout.

Sample size was calculated based on the rate of surgery cancellations for medical reasons (2%),^16^ with a 5% alpha and a margin of error of 1.1%, resulting in a required sample of 622 patients. An additional 5% was added to account for potential losses to follow up, yielding a final sample size of 700 patients.

Selection was by convenience sampling. Univariate and multivariate analyses identified factors associated with preoperative and intraoperative complications. Descriptive results are shown in tables using measures of central tendency and dispersion for numerical variables, and frequencies for categorical ones. Data normality was tested using the Shapiro-Wilk test, with results expressed as mean ± standard deviation or median (interquartile range).

To assess Systolic (SBP), Diastolic (DBP), and Heart Rate (HR) variations across three time points, repeated-measures ANOVA with Bonferroni correction or Friedman’s test with Dunn’s post hoc was used, depending on normality. Associations between complications and ASA status, hypertension, and heart disease were tested using Chi-Square or Fisher's exact test. Binary logistic regression identified associations between patient characteristics and complications. A 5% significance level was adopted. Statistical analysis was performed using SPSS v26.

## Results

A total of 700 Urology Department patients were included. Table 1 summarizes baseline characteristics, and Table 2 reports comorbidities identified during outpatient consultations. The total number of comorbidities is 1,048, as some patients had more than one condition, so cumulative frequency exceeds participants. The most prevalent were hypertension (53.7%), tobacco use (40.6%), cardiovascular disease (17.7%), and diabetes mellitus (14.3%). Patients with any comorbidity were treated and stabilized in the outpatient setting and returned for preanesthetic reassessment and surgical clearance. A total of 376 hypertensive patients were identified; some had long-standing hypertension, others were newly diagnosed. All initiated antihypertensive treatment and remained on therapy until surgery, maintaining normal blood pressure. These factors influenced risk stratification, anesthetic management, and preoperative optimization. Table 3 shows variations in blood pressure and heart rate across outpatient, preoperative, and intraoperative periods for ASA I, II, and III physical status groups. Systolic and diastolic pressures significantly decreased from outpatient to preoperative and intraoperative phases in all ASA groups (p < 0.0001). Heart rate also decreased intraoperatively in ASA I (p = 0.0012) and III (p = 0.0010) patients.

**Table 1:** Baseline characteristics of the study participants.

Variable	Results (n = 700)					
**Sex, n (%)**						
Male	627 (89.6)					
Female	73 (10.4)					
**Color, n (%)**						
White	415 (59.3)					
Brown	201 (28.7)					
Black	84 (12.0)					
**ASA, n (%)**						
I	92 (13.1)					
II	426 (60.9)					
III	182 (26.0)					
	**Mean**	**SD**	**Median**	**IIQ**	**Minimum**	**Maximum**
**Age (years)**	64.2	12.7	67	59‒73	18	93
**Weight (kg)**	70.1	11.7	69	62‒78	42	114
**Height (cm)**	166.6	6.7	166	162‒170	148	190

ASA, Physical Status Classification. Numerical data expressed as mean ± standard deviation; categorical data expressed as frequency (percentage) IIQ, Interquartile range (Q1‒Q3).

**Table 2:** Comorbidities and clinical risk factors detected in preanesthetic evaluation.

Variable	Results (n = 1048)^a^
Hypertension	376 (53.7)
Tobacco use disorder	284 (40.6)
Heart disease	124 (17.7)
Diabetes mellitus	100 (14.3)
Obesity	60 (8.6)
Lung disease	53 (7.6)
Anxiety	29 (4.1)
Cancer	22 (3.1)

Data presented as frequency (percentage).

a The same patient could present more than one comorbidity and risk factor.

**Table 3:** Blood pressure and heart rate by ASA class and perioperative timepoint.

	Outpatient	Preoperative	Intraoperative	p-value
**ASA I (n = 92)**				
SBP (mmHg)	129.1 ± 14.6^a^	127.1 ± 15.9^a^	116.3 ± 14.9^b^	< 0.0001
DBP (mmHg)	80.7 ± 9.8^a^	77.6 ± 9.4^a^	73.1 ± 10.7^b^	< 0.0001
HR (bpm)	75.9 ± 10.0^a^	75.7 ± 9.1^a,b^	73.2 ± 8.9^b^	0.0012
**ASA II (n = 426)**				
SBP (mmHg)	143.8 ± 19.8^a^	139.3 ± 19.5^b^	124.0 ± 19.9^c^	< 0.0001
DBP (mmHg)	86.5 ± 11.4^a^	82.9 ± 10.7^b^	76.2 ± 12.14^c^	< 0.0001
HR (bpm)	76.3 ± 10.6	76.04 ± 9.5	75.29 ± 9.2	0.2270
**ASA III (n = 182)**				
SBP (mmHg)	143.2 ± 18.7^a^	139.6 ± 19.7^a^	124.8 ± 21.4^b^	< 0.0001
DBP (mmHg)	86.8 ± 11.0^a^	82.4 ± 10.1^b^	74.8 ± 12.7^c^	< 0.0001
HR (bpm)	77.6 ± 10.5^a^	75.3 ± 4.4^b^	74.4 ± 8.9^b^	0.0010

SBP, Systolic Blood Pressure; DBP, Diastolic Blood Pressure; HR, Heart Rate; ASA, Physical Status Classification. Data presented as mean ± standard deviation. Different letters denote a significant difference (p < 0.05; repeated measures ANOVA test, with Bonferroni post-test).

Different letters indicate statistically significant differences. In ASA I patients, both Systolic (SBP) and Diastolic Blood Pressure (DBP) were significantly lower intraoperatively compared to the outpatient and preoperative periods. Heart rate was also lower intraoperatively compared only to the outpatient measurement. In ASA II, SBP and DBP were lower intraoperatively than in both outpatient and preoperative periods, with preoperative values also lower than outpatient. In ASA III, SBP was lower intraoperatively compared to both outpatient and preoperative periods. DBP followed the same pattern, with preoperative values also lower than outpatient. Finally, heart rates in both preoperative and intraoperative periods were lower than in the outpatient setting.

Of the 700 patients, 650 were over 40 years old and underwent Electrocardiography (ECG). Sinus rhythm was observed in 341 patients (52.5%), while abnormalities were present in 47.5%. The most frequent findings were extrasystoles (18.2%), left ventricular hypertrophy (12.5%), right bundle branch block (8.4%), and signs of acute myocardial infarction (8.2%). Ninety-one patients with ECG abnormalities and suggestive clinical signs were referred for echocardiography. Structural or functional alterations were identified in 69 cases (10.6%), including hypertensive heart disease (3.5%), ischemic heart disease (3.1%), ventricular dysfunction (2%), diastolic dysfunction (1.4%), and mitral insufficiency (0.6%).

The main surgeries performed were Transurethral Resection (TUR) of the prostate (52.3%) and bladder tumor (6.9%), followed by pyelolithotomy (4.7%), nephrectomy (4.6%), radical prostatectomy (4.1%), and suprapubic prostatectomy (4.1%). Additional surgeries included orchiectomy, cystopexy, hydrocelectomy, and transgenitalization.

Spinal anesthesia was the predominant technique (77.9%), followed by general anesthesia (21%), epidural (9.4%), sedation (1%), and local anesthesia (0.7%). These percentages also account for cases in which anesthetic techniques were combined.

Table 4 presents preoperative and intraoperative complications according to ASA physical status. The preoperative findings refer to events observed upon entry into the operating room, during monitoring, before any anesthetic technique was initiated. This includes both patients with a previous history of hypertension and those without such a history who experienced a transient elevation in systemic blood pressure immediately prior to surgery.

**Table 4:** Preoperative and intraoperative complications according to ASA physical status classification.

Variable	ASA I (n = 92)	ASA II (n = 426)	ASA III (n = 182)	p-value
Preoperative				
Hypertension	1 (1.1)	12 (2.8)	1 (0.5)	0.1599
Atrial fibrillation	0 (0.0)	0 (0.0)	1 (0.5)	0.2405
Intraoperative				
Hypotension	4 (4.3)	36 (8.5)	17 (9.3)	0.3371
Hypertension	0 (0.0)	12 (2.8)	4 (2.2)	0.2597
Bradycardia	1 (1.1)	6 (1.4)	1 (0.5)	0.6584

ASA, American Society of Anesthesiologists physical status classification. Data presented as frequency (percentage).

Preoperative monitoring in the operating room revealed hypertension (2.0%), anemia (0.6%), atrial fibrillation (0.1%), and bronchospasm (0.1%). One case, a 77-year-old male (ASA II) with hypertension and heart disease, had surgery canceled due to severe blood pressure elevation (200/130 mmHg). Intraoperative complications included hypotension (8.1%), inadequate neuraxial block (2.6%), hypertension (2.3%), bradycardia (1.1%), dura mater perforation (0.1%), and allergic reactions (0.7%).

No preoperative or intraoperative deaths occurred. Hemodynamic parameters progressively declined from the outpatient to intraoperative periods across ASA groups. Both systolic and diastolic blood pressures decreased significantly (p < 0.0001), while heart rate declined significantly in ASA I and ASA III patients. These changes likely reflect anesthetic effects rather than instability. Even in ASA III, reductions were not excessive, suggesting effective preoperative optimization. Structured preanesthetic consultations may be associated with intraoperative stability by addressing modifiable risks, supporting the value of comprehensive assessment, especially in high-risk patients.

Binary logistic regression identified independent risk factors for preoperative and intraoperative complications, as shown in Table 5. Outpatient SBP (p = 0.029), preoperative SBP (p < 0.001), preoperative DBP (p = 0.001), and intraoperative heart rate (p = 0.026) were associated with an increased risk of preoperative complications. Additionally, age (p = 0.008) and preoperative SBP (p = 0.029) were associated with a higher risk of intraoperative complications. In contrast, intraoperative SBP (p < 0.001), DBP (p < 0.001), and heart rate (p < 0.001) were protective factors against intraoperative complications.

**Table 5:** Multivariate logistic analysis to evaluate associations between patient characteristics and complications.

Patient characteristics	Preoperative adverse events			Intraoperative adverse events		
	OR	95% CI	p-value	OR	95% CI	p-value
Sex^a^	0.608	0.079‒4.693	0.633	0.701	0.294‒1.676	0.425
Color^b^	1.840	0.230‒14.719	0.566	2.802	0.984‒7.976	0.054
ASA	0.845	0.367‒1.945	0.692	1.270	0.860‒1.876	0.229
Age	1.010	0.968‒1.055	0.640	1.031	1.008‒1.054	0.008^c^
Weight	0.974	0.929‒1.021	0.276	0.992	0.972‒1.013	0.461
Height	0.963	0.891‒1.040	0.336	0.979	0.944‒1.014	0.235
Outpatient SBP	1.028	1.003‒1.054	0.029^c^	1.008	0.996‒1.021	0.169
Outpatient DBP	1.002	0.957‒1.048	0.943	1.013	0.992‒1.034	0.222
Outpatient HR	0.988	0.941‒1.038	0.638	1.005	0.982‒1.027	0.693
Preoperative SBP	1.062	1.035‒1.088	< 0.001^c^	1.013	1.001‒1.025	0.029^c^
Preoperative DBP	1.072	1.027‒1.118	0.001^c^	1.008	0.986‒1.031	0.472
Preoperative HR	1.018	0.963‒1.077	0.525	1.012	0.986‒1.038	0.377
Intraoperative SBP	1.018	0.994‒1.043	0.139	0.961	0.948‒0.974	< 0.001^c^
Intraoperative DBP	1.018	0.976‒1.061	0.406	0.941	0.922‒0.961	< 0.001^c^
Intraoperative HR	1.073	1.008‒1.141	0.026^c^	0.904	0.879‒0.931	< 0.001^c^

SBP, Systolic Blood Pressure; DBP, Diastolic Blood Pressure; HR, Heart Rate; Reference variables were those that presented the highest frequency.

a Male sex as a reference variable.

b White color as a reference variable.

c Significant association (p < 0.05).

## Discussion

In this retrospective study of 700 patients undergoing urological procedures, outpatient preanesthetic consultation conducted in advance played a key role in improving patient preparation and perioperative outcomes. Early evaluation allowed time for diagnostic testing, clinical optimization, and, when needed, multidisciplinary input. It also enabled individualized anesthetic planning, early risk identification, and reduced surgical cancellations. This structured approach enhances perioperative safety and supports efficient, patient-centered care.

In contrast, same-day evaluations tend to be brief and reactive, focused on excluding immediate contraindications, which limit comprehensive assessment, risk stratification, and preventive or optimization strategies. According to the ASA’s Practice Advisory for Preanesthesia Evaluation, assessments should preferably occur within 30 days before surgery and be updated within 48 hours, especially for high-risk or complex cases.^10^

In recent years, telemedicine has emerged as promising strategy for outpatient preanesthetic evaluations, particularly for ASA I and II patients, in accordance with ASA guidelines. The ASA emphasizes that remote consultations must maintain core elements such as clinical history, physical examination when feasible, and informed consent.^17^ Studies such as Kamdar et al.^11^ have demonstrated the safety, feasibility, efficiency, and patient satisfaction of this model in major academic centers. In Brazil, Machado et al.^12^ and Freitas^13^ reported similar positive outcomes in public hospitals, noting improved access, reduced costs, and no increase in surgical cancellations. These findings support telemedicine as a viable alternative for selected low-risk patients.

The most prevalent chronic conditions identified during outpatient consultations were hypertension, smoking, heart disease, diabetes, and obesity, all known to significantly increase perioperative risk. These findings are consistent with Klopfenstein et al.^18^ and Zambouri,^19^ who also reported high rates of hypertension, obesity, and tobacco use in surgical populations, reinforcing the importance of early identification and management of chronic conditions.

Hypertension was diagnosed in 53.7% of patients during the preanesthetic evaluation, aligning with prevalence rates of around 60% in individuals over 60, as reported by Dix et al.^20^ and De Paula.^21^ This is consistent with mean age of 64.2 years in this study. Early identification enabled therapeutic adjustments that may be associated with better perioperative management and surgical outcomes.

Patients received individualized care and anesthesia-focused guidance in outpatient setting. This facilitated early detection and control of hypertension. As a result, significant reduction in preoperative blood pressure was observed across all ASA classes (I, II, III) compared to outpatient values. Intraoperative heart rate also significantly decreased in ASA I and III patients. These results are consistent with Shirdel et al.,^22^ who reported that qualified preoperative counseling and continued care until admission led to reductions in anxiety, systolic blood pressure, heart rate, and respiratory rate.

In the present study, causal inferences regarding the effect of preanesthetic consultation on blood pressure reduction cannot be drawn in absence of control group. Our project was to characterize the clinical trajectory of patients following the preanesthetic consultation rather than perform comparative analyses. Reductions in blood pressure were observed both upon entry into operating room and during intraoperative period; nevertheless, these findings warrant cautious interpretation. Preoperative changes may reflect the combined influence of pharmacologic optimization, preanesthetic medication, perioperative anxiolysis, or other contextual factors. Accordingly, conclusions were framed to reflect descriptive and observational design of study, avoiding unwarranted causal attribution.

Elective surgeries are often canceled due to uncontrolled hypertension, particularly in older adults, as noted by Dix et al.^20^ and De Paula.^21^ However, in this study, only one surgery was canceled, suggesting early outpatient evaluation allowed timely intervention and effective risk management. This emphasizes the role of preanesthetic assessment in clinical optimization, improving surgical scheduling, and reducing preventable cancellations.

Several studies, including Fischer et al.,^2^ Kristoffersen et al.,^3^ Tait et al.,^23^ and Rosner,^24^ show preanesthetic consultations reduce delays, cancellations, hospital costs, unnecessary tests, and complications. Among 700 scheduled procedures in this study, only one was canceled, reflecting the effectiveness of outpatient evaluations. Early consultations help identify and manage clinical issues in advance, enhancing care coordination and minimizing avoidable delays.

Conversely, Epstein et al.^25^ reported high cancellation rates when patients were assessed only the night before surgery. Similarly, Zambouri^19^ found many patients were examined only minutes before surgery. These scenarios highlight the benefits of a proactive, structured preanesthetic approach. In this study, patients were initially assessed in the outpatient clinic and re-evaluated the day before surgery by anesthesiology residents, who also prescribed preanesthetic medications as needed.

ECGs were performed in patients aged 40 and older, with 47.5% showing abnormalities, supporting findings from Alanzy et al.,^26^ Correll et al.,^27^ and Mossie et al.^28^ regarding increased cardiovascular risk in older adults. Echocardiograms were requested only when ECG abnormalities were accompanied by clinical signs, aligning with Kristoffersen et al.^3^ Fischer et al.^2^ noted that preanesthetic assessments led to fewer unnecessary tests compared to surgeon-managed preoperative care. At HUCFF, tests were requested by surgeons but followed ASA-based recommendations jointly established with the Anesthesiology Outpatient Clinic, ensuring a standardized, clinically appropriate approach.

This study was carried out in a teaching hospital, where anesthesiology residents and medical students assumed a central role in outpatient consultations. This model aligns with Fischer,^2^ who highlights the importance of active resident involvement in preanesthetic evaluations.

This study has limitations. Being conducted at a single center may limit external validity. Total surgical duration was not recorded, and the exact timing of intraoperative complications was not documented, preventing analysis of associations with specific phases or procedure length. The retrospective design and institutional constraints precluded inclusion of a control group or comparison with patients evaluated by other specialties or only on the day of surgery. Consequently, findings should be interpreted as exploratory, and observed blood pressure reductions may reflect multiple factors, including pharmacologic optimization, preanesthetic medication, perioperative anxiolysis, or clinical course. Future prospective comparative studies with control groups and standardized protocols are needed to confirm these observations and clarify their clinical significance.

In Brazil, where structural inequalities affect access to quality of healthcare, particularly in public system (SUS), cost-effective strategies that improve patient experience and optimize preoperative conditions are especially valuable. Preanesthetic consultation enables clinical assessment, builds trust, reduces anxiety, and enhances patient safety. In public healthcare, where time constraints and limited follow-up are common, this approach supports quality care, equity, and helps mitigate disparities.

## Conclusion

The study population consisted mainly of older adults, with a high prevalence of chronic conditions such as hypertension, tobacco use, heart disease, diabetes, and obesity. Cardiovascular abnormalities, particularly ECG changes, were commonly detected during outpatient consultations, emphasizing the importance of early risk identification and timely treatment. Reductions in preoperative and intraoperative systolic and diastolic blood pressure were observed compared with outpatient values, while intraoperative heart rate was also significantly lower. Only one surgery was canceled due to hypertension. Preoperative and intraoperative complications were infrequent and manageable, suggesting early assessment helps prevent complications and promotes intraoperative stability.

Reductions in systolic and diastolic blood pressure were documented upon operating room entry among patients evaluated in outpatient preanesthetic consultation, with a very low rate of surgical cancellations, although further comparative and prospective studies are needed. This approach may also support better perioperative outcomes by enabling early risk identification, individualized planning, and reduced cancellations. Additionally, standardized test protocols based on clinical criteria, rather than those defined solely by surgical teams, proved appropriate and efficient.

In a healthcare system with structural inequalities and limited resources, such as Brazil’s public system, cost-effective strategies optimizing preoperative preparation and enhancing patient experience are particularly relevant. These findings support broader implementation of outpatient preanesthetic assessments to improve surgical care quality and system efficiency.

## Artificial intelligence use statement

The authors declare that an artificial intelligence tool [ChatGPT] was used exclusively for text organization. No AI tool was used for data analysis or interpretation of the results. The authors take full responsibility for the content of the manuscript.

## Data availability statement

The datasets generated and/or analyzed during the current study are available from the corresponding author upon reasonable request.

## Conflicts of interest

The authors declare no conflicts of interest.
